# Assessment of the efficacy of a topical combination of fipronil-permethrin (Frontline Tri-Act^®^/Frontect^®^) against egg laying and adult emergence of the cat flea (*Ctenocephalides felis*) in dogs

**DOI:** 10.1051/parasite/2016068

**Published:** 2016-12-19

**Authors:** Frédéric Beugnet, Lénaïg Halos, Wilfried Lebon, Julian Liebenberg

**Affiliations:** 1 Merial S.A.S. 29 Av. Tony Garnier 69007 Lyon France; 2 ClinVet International (Pty) Ltd. Uitsig Road, Bainsvlei 9321 Bloemfontein South Africa

**Keywords:** Dogs, Fleas, *Ctenocephalides felis*, Frontline Tri-Act^®^/Frontect^®^, Fipronil, Permethrin, Egg laying

## Abstract

This study was conducted to assess the prevention of egg laying and the inhibition of the emergence of the cat flea (*Ctenocephalides felis*) resulting from the application of a combination of fipronil and permethrin (Frontline Tri-Act^®^/Frontect^®^, Merial) on dogs. Sixteen healthy dogs were included after pre-treatment live flea counts and randomly allocated to two groups. Eight dogs served as untreated controls and 8 dogs were treated on Day 0 and Day 30 with topical application of fipronil/permethrin at the minimum dose of 6.76 mg/kg fipronil and 50.48 mg/kg permethrin. On days −2, 7, 21, 28, 42 and 56, each dog was infested with 100 fleas. Flea eggs were collected from each dog in individual trays from 12 to 36 h after treatment or each flea re-infestation. All fleas were removed by combing and counted 36 h after treatment or infestations. The collected eggs were counted and incubated for 28 days for larval development and adult emergence assessment. The curative efficacy of Frontline Tri-Act^®^/Frontect^®^ against adult fleas 36 h after treatment was 95.3% and the efficacy remained 100% after subsequent flea infestations for 8 weeks. Compared to the control group, the treatment reduced egg laying by 84.5% within 36 h after first treatment and was 99.9%, 100%, 100%, 100%, 100% on collection days 7, 21, 29, 43 and 57, respectively. Frontline Tri-Act^®^/Frontect^®^ reduced by 28.7% the emergence of new adult fleas from eggs laid during the 48 h of pre-treatment infestation. The inhibition of adult emergence from incubated flea eggs could not be assessed after flea re-infestation in the treated group as no eggs were collected.

## Abstract (fr)

Cette étude a été conduite pour estimer la réduction de production d’œufs de puces ainsi que l’inhibition d’émergence de nouvelles puces du chat (*Ctenocephalides felis*) suite au traitement de chiens par application de la combinaison de fipronil et de perméthrine (Frontline Tri-Act^®^/Frontect^®^, Merial). Seize chiens sains ont été inclus et répartis de façon aléatoire en deux groupes après infestation et comptage de puces avant traitement. Huit chiens ont servi de contrôles non traités tandis que 8 chiens ont été traités aux jours 0 et 30 par application topique (« spot on ») d’une formulation de fipronil/perméthrine à la dose minimale de 6.76 mg/kg de fipronil et 50.48 mg/kg de perméthrine. Aux jours −2, 7, 21, 28, 42 et 56 chaque chien a été infesté par 100 puces. Les œufs de puces ont été récoltés sur des plateaux à partir de chaque chien mis en cage individuelle entre 12 et 36 heures après traitement ou après chaque infestation. Toutes les puces ont été retirées par peignage et comptées au terme de ces 36 heures post traitement ou infestation. Les œufs collectés ont été comptés puis incubés durant 28 jours de façon à obtenir un développement larvaire et à estimer l’émergence de nouvelles puces. L’efficacité curative à 36 heures post-traitement de Frontline Tri-Act^®^/Frontect^®^ vis-à-vis des puces adultes étaient de 95.3 % et l’efficacité préventive vis-à-vis des infestations hebdomadaires est restée à 100 % durant 8 semaines. Comparé au groupe contrôle, le traitement a réduit la production d’oeufs de puces de 84.5 % 36 heures après application, puis a réduit la production d’œufs de 99.9 %, 100 %, 100 %, 100 %, 100 % pour les collectes des jours 7, 21, 29, 43 et 57, respectivement. Frontline Tri-Act^®^/Frontect^®^ a réduit de 28.7 % l’émergence de nouvelles puces à partir des oeufs issus des puces ayant été déposées sur les chiens 48 heures avant traitement. L’inhibition de l’émergence de nouvelles puces n’a pu être étudiée à partir des ré-infestations de puces dans le groupe traité puisqu’aucun œuf n’a été récolté.

## Introduction

The cat flea *Ctenocephalides felis* (Bouché, 1835) is the predominant ectoparasite of dogs and cats worldwide, representing at least 80% of the flea infestations in Europe [12, 13]. Fleas are competent vectors of flea-borne pathogenic agents with zoonotic potential such as *Bartonella henselae*, agent of cat scratch disease, and *Rickettsia felis*, the causative agent of flea-borne spotted fever. Fleas are also the intermediate host of the dog and cat tapeworm *Dipylidium caninum* [1, 13, 15]. Depending on individual factors and the number of fleas feeding on the host, flea bites could lead to pruritus, hairloss, skin lesions, and flea allergic dermatitis (FAD), which is the most common dermatological disease in dogs [5, 6, 17, 18].

One of the major difficulties in flea control and a challenge for veterinarians is the awareness of owners about flea biology and resulting disease (life-cycle, identification of infestation), and their compliance with treatment and prevention of infestations [4, 12, 13]. Once fleas are on the host, they start feeding within a few minutes, then mate in the following hours and start producing eggs in the 24–36 h following the first blood meal [6, 18]. Female fleas lay eggs in the hair coat (up to 50 eggs/day) that fall from the pelage in the host environment where they hatch and develop into adults in a few weeks [6, 17, 18]. The critical issue is that only 5% of the flea population is represented by adults while immature stages remain the non-visible part spread in the environment [4, 13]. Integrated flea management includes control of both adult and immature stages. Successful flea control on pets is based on killing adult fleas on hosts, breaking the life-cycle, and controlling the environmental contamination. Anti-flea products containing insect growth regulators (IGRs) have proven to be effective for the control of infested environments [4, 13, 17]. Without IGRs, it has been demonstrated recently with ectoparasiticides containing afoxolaner, fluralaner or sarolaner, that a sustained high speed of kill can prevent egg production and thus avoid environmental contamination by immature flea stages [3, 7, 8, 19, 20].

Frontline Tri-Act^®^/Frontect^®^ spot-on solution for dogs (Merial) is a combination of fipronil (6.76 mg/kg) and permethrin (50.48 mg/kg) designed to kill fleas and ticks, and to repel sandflies, mosquitoes and stable flies. A single topical application provides immediate and sustained insecticidal efficacy against adult fleas [11]. Recently, the speed of kill provided by Frontline Tri-Act^®^ against *C. felis* and *Ctenocephalides canis* fleas was demonstrated, with a sustained killing effect in 6 h for 4 weeks against new infesting fleas [2, 14].

The present study intended to assess the prevention of egg laying provided by Frontline Tri-Act^®^ against existing and new infesting fleas, and therefore the inhibition of adult flea emergence in order to confirm that the rapid speed of kill prevents environmental contamination by flea eggs.

## Materials and methods

This study was designed in accordance with the guidelines of the European Committee for Veterinary Medicinal Products (EMA-CVMP) for the testing and evaluation of the efficacy of antiparasitic substances for the treatment and prevention of tick and flea infestation in dogs and cats [10], and the guidelines of the World Association for the Advancement of Veterinary Parasitology (WAAVP) [16]. It complied with Good Clinical Practices as described in the International Cooperation on Harmonisation of Technical Requirements for Registration of Veterinary Medicinal Products, VICH Guideline 9 [9]. This study was conducted at ClinVet Contract Research Organisation, Bloemfontein, in South Africa.

### Animals

Healthy, purpose-bred laboratory dogs (mixed breeds and Beagles) of both sexes were included. They had not been treated with a long-acting acaricide/insecticide (either topical or systemic) during the 12 weeks preceding Day 0. Dogs had also not been treated with compounds containing IGRs within the past 6 months. Twenty dogs were acclimatised to the study conditions. On Day −9, a pre-treatment flea infestation was performed and count was conducted on Day −6 to evaluate the receptivity of each dog to experimental infestation and for random allocation to the groups. Dogs were then ranked within sex in descending order of individual pre-treatment live flea counts and the four dogs with the lowest live flea count were removed. Sixteen dogs (7 males and 9 females) weighing 10.4–25 kg were included and randomly allocated to the untreated control group (8 dogs) or to the Frontline Tri-Act^®^/Frontect^®^ treated group (8 dogs).

Animals were kept individually in cages with concrete floors and fitted with a sleeping bench. On Days −7, 0, 7, 21, 28, 42 and 56 dogs were enclosed in stainless steel cages with mesh floors and trays in order to collect flea eggs. On these days, all the dogs were enclosed in the stainless steel cages from hour 12 post treatment or post flea infestation until hour 36 (i.e. for a period of 24 consecutive hours). No physical contact between dogs was possible. Dogs were fed once a day with standard commercially available diets. Potable water was provided in stainless steel bowls and replenished at least twice daily.

All animals were managed similarly with due regard for their well-being and in compliance with the Merial Ethics Committee and local applicable animal welfare regulations and requirements (South African National Standard SANS 10386:2008 “The care and use of animals for scientific purposes”).

### Study design

This efficacy study was conducted under a negative controlled and blinded design. Animals were acclimatised to the study conditions 9 days prior to treatment. A physical examination performed by a veterinarian during the acclimation confirmed that they were clinically healthy. To detect any treatment-related or unrelated adverse events, health observations were conducted at least once daily from the start of acclimation to the end of the study. In addition, dogs were observed at hourly intervals for 4 h after each treatment.

A European laboratory bred strain of *C. felis* (routinely fed on cats) was used for all infestations.

Dogs allocated to the control group remained untreated (Group 1). On Day 0 and Day 30, dogs from Group 2 received a topical application of fipronil/permethrin (Frontline Tri-Act^®^/Frontect^®^, Merial) at a dose of 0.1 mL/kg (based on bodyweight) equivalent to 6.76 mg/kg fipronil and 50.48 mg/kg permethrin using an appropriate sized syringe without a needle. The second treatment, conducted at Day 30, was done after the egg collection and the flea comb (ending at Day 29). The treatment was applied topically as described in the label, directly onto the skin, through parting the hair until the skin was visible, in two spots at the base of the neck in front of the shoulder blades and in the middle of the neck between the base of the skull and the shoulder blades.

Dogs were infested with 100 adult unfed fleas on Day −9 for allocation purposes, and on Days −2, 7, 21, 28, 42 and 56 to assess efficacy. All live fleas remaining on the dog were removed and counted via thorough combing of the full body areas with a fine-tooth flea comb on Day 1 (36 ± 2 h after treatment) and then 36 ± 2 h after each of the subsequent weekly flea infestations. Live flea counts were always performed after egg collections.

Eggs laid from fleas of each infested dog were collected during a period of 24 h, from 12 to 36 h after treatment or flea re-infestations. For the flea egg collection, each dog was placed in a steel cage over a tray. The tray was covered by a paper liner where the eggs could fall. In order to avoid soiling, a first tray was used during a 12 h period and then replaced by a second from 24 h to 36 h. The total number of eggs collected per dog per each flea infestation was calculated by adding the two 12 h collections. After counting, a maximum of 50 eggs per collection period of 12 h from individual dogs were placed in Petri dishes containing flea growth medium and placed in humidity containers in a 25 °C temperature-controlled room. Eggs were incubated for 28 days for adult flea emergence assessments on Days 29, 36, 50, 57, 71 and 85. Like for the egg count, the adult flea emergence was calculated by adding the adults obtained from the two successive collection periods per each flea infestation.

### Data analysis

Data were analysed separately using SAS version 9.3. Based on the EMEA/CVMP/005/2000-Rev. 2 guideline [10], the primary efficacy calculations were based on arithmetic mean values with geometric mean values considered secondary.

The differences between the treated group and the control group were analysed statistically by means of an analysis of variance (ANOVA) with a treatment effect on both untransformed and logarithmic transformed data. All testing was two-sided at the significance level of *p* = 0.05.

#### Prevention of egg laying

The percent reduction in egg laying (primary criteria) in the treated group compared to the control group was calculated for each time point using the formula:

% Egg laying prevention=100×[total number of collected eggs (Control) –total number of collected eggs (Treated)]/total number of collected eggs (Control)

#### Adult flea emergence inhibition

The percent reduction of adult emergence (secondary criteria) in the treated group compared to the control group was calculated using the mean number of emerged adult fleas divided by the mean number of eggs collected and incubated at each time point.

% Inhibition of adult flea emergence = 100×[proportion of emerged adults (Control) - proportion of emerged adults (Treated)]/proportion of emerged adults (Control)

Proportion of emerged adults = Number of emerged adult fleas/number of incubated eggs in the group.

If no eggs were collected from any animal on a given day, the proportion of emerged adult fleas was defined as zero for the purposes of the calculations of the arithmetic means.

#### Adulticidal efficacy

The curative efficacy (against existing fleas) and persistent efficacy (against new infestations) were determined from live flea counts in the treated group compared to the control group following the formula:

% Efficacy=[Arithmetic mean of flea counts (Control)- Arithmetic mean of flea counts (Treated)]/Arithmetic mean of flea counts (Control)

At Day 2, the curative efficacy against existing fleas was assessed, whereas preventive efficacy against new infesting fleas was assessed after each subsequent infestation.

## Results

Following the WAAVP guideline [16], the study was considered valid as all control dogs demonstrated adequate flea retention rates (>50%) during the study with means of 84.4 to 94.5 fleas on the untreated control dogs.

One female dog of the treated group was removed from the study on Day 30 following confirmation of a carcinoma of the mammary gland, which was considered not related to the treatment.

The egg production for both groups is summarised in Table 1. Arithmetic means of egg counts in the control group ranged from 140.9 to 712.4. The egg production was reduced by 84.5% within 36 h after first Day 0 treatment on dogs infested on Day −2. The prevention of egg laying after Day 0 against new flea challenges was 99.9%, 100%, 100%, 100% and 100% on days 8, 22, 29, 43 and 57, respectively. The egg counts in the fipronil/permethrin group remained significantly different throughout the study from the control dogs (*p* < 0.05).

**Table 1.** Flea egg counts and prevention of flea egg laying (%) using arithmetic means.

Day	Egg counts	Egg counts (egg laying prevention %)	*p*-value
	Control group 1	Treated group 2	
Day 1	712.4	110.3 (84.5%)	0.0025
Day 8	140.9	0.1 (99.9%)	0.0034
Day 22	266.1	0.0 (100.0%)	0.0023
Day 29	239.4	0.0 (100.0%)	0.0003
Day 43	246.9	0.0 (100.0%)	0.0018
Day 57	261.4	0.0 (100.0%)	0.0002

*p*-value: One-way ANOVA with a treatment effect.

Group 1: Negative control.

Group 2: Dogs were treated topically with Frontline^®^ Tri-Act at the minimum dose of 0.1 mL/kg.

Adult emergence inhibition, as defined by the number of viable adult fleas that emerged following a 28-day incubation period, is summarised in Table 2. In the control group, 53.5%– 72.2% of incubated eggs hatched and larvae evolved into adults (arithmetic means of new fleas 42–58). The efficacy of Frontline Tri-Act^®^/Frontect^®^ was 28.7% regarding eggs originating from the existing flea infestation (Day −2) collected on Day 0 to 1, which was not significantly different from the control. Thereafter, no eggs were collected; therefore, the inhibition of emergence cannot be studied in the treated group.

**Table 2.** Proportion of new fleas obtained from eggs and inhibition of adult flea emergence using arithmetic means.

Collection day	Mean proportion of new fleas from eggs	Mean proportion of new fleas from eggs (inhibition of new flea emergence %)	*p*-value
	Group 1 (control)	Group 2 (treated)	
Day 1	0.533	0.380 (28.7%)	0.2302
Day 8	0.685	NA*	
Day 22	0.597	NA*	
Day 29	0.722	NA*	
Day 43	0.663	NA*	
Day 57	0.660	NA*	

*p*-value: One-way ANOVA with a treatment effect.

Group 1: Negative control.

Group 2: Dogs were treated topically with Frontline^®^ Tri-Act at the minimum dose of 0.1 mL/kg.

* Not Applicable because of the absence of flea eggs.

The adulticidal efficacy, as assessed by live flea counts, is summarised in Table 3. Frontline Tri-Act^®^/Frontect^®^ provided 95.3% efficacy against pre-existing flea infestation. The sustained efficacy was then 100% after each new infestation.

**Table 3.** Flea counts on dogs and 36h efficacy based arithmetic means.

Day	Mean flea counts	Mean flea counts (Eff.%)	*p*-value
	Group 1 (control)	Group 2 (treated)	
Day 1	84.4	4.0 (95.3%)	<.0001
Day 8	88.4	0.0 (100.0%)	<.0001
Day 22	94.5	0.0 (100.0%)	<.0001
Day 29	86.6	0.0 (100.0%)	<.0001
Day 43	86.5	0.0 (100.0%)	<.0001
Day 57	93.0	0.0 (100.0%)	<.0001

*p*-value: One-way ANOVA with a treatment effect.

Group 1: Negative control.

Group 2: Dogs were treated topically with IVP Frontline^®^ Tri-Act at the minimum dose of 0.1 mL/kg.

## Discussion

The goal of the present study was to determine whether the sustained speed of kill of Frontline Tri-Act^®^/Frontect^®^ could prevent the production of flea eggs and thus break the flea life-cycle. The treatment quickly stopped egg production of existing female fleas present on dogs, with a curative efficacy of 84.5% in 36 h. It almost completely prevented the production of eggs by new infesting fleas during the first month (>99.9%), and completely (100%) during the second month. These results confirm the high level of adulticidal efficacy of Frontline Tri-Act^®^/Frontect^®^ against both existing and new infesting fleas, as previously reported for this product [2, 14].

Female fleas start producing eggs as early as 12 h after infestation but with regular production after 24 h [18], which was observed throughout the study in the control group. The sustained speed of kill of an insecticidal product is essential for the control of environmental contamination by immature flea stages. The ability to prevent egg production has recently been demonstrated in experimental studies with ectoparasiticides containing isoxazolines (afoxolaner, sarolaner or fluralaner) having a sustained speed of kill of less than 24 h for 1–3 months [3, 19, 20]. It has also been demonstrated in the field in studies using flea traps to assess environmental control [7, 8]. Halos et al. [14] and Beugnet et al. [2] recently demonstrated the rapid onset of action and the knock-down effect of Frontline Tri-Act^®^/Frontect^®^ on *C. felis* and *C. canis* fleas. Efficacy was >98.5% (*C. felis*) and >99.1% (*C. canis*) within 6 h after infestation for 28 days [2, 14]. In the present study, the number of eggs produced on Day 0 to 1 in both groups was related to the pre-treatment infestation that occurred 48 h before the topical application. The egg count was significantly lower in the treated group than in the control group (110.3 and 712.4, respectively), indicating rapid death of the mature gravid fleas. It is known that the active ingredients (i.e. fipronil and permethrin) act by contact after topical application and need to diffuse all over the dog’s body, which takes from 24 to 48 h [11, 13]. Looking at the emergence of new adult fleas from these collected eggs at Day 0 to 1, the proportion of emergence was 0.535 and 0.380 in the control and treated group, respectively. The 28.7% inhibition in the treated group compared to the control group was not significant. It cannot be concluded that there is any direct activity of the combination on the evolution of eggs. The direct action on eggs is the principal mode of action of insect growth regulators like (S)-methoprene or pyriproxyfen, which inhibit >90% of immature stage evolution [4, 13]. On Day 7, only one egg was collected from a treated dog but it did not evolve into an adult flea. Afterwards, no eggs were collected from treated dogs and consequently no adult fleas emerged. In the present study, the emergence of new fleas ranged from 53% to 66% in the untreated control group, but emergence could not be assessed from the egg stage in the treated group. The absence of new adult fleas from the treated dogs was a direct consequence of the absence of eggs.

This study demonstrated that with monthly application, the speed of kill of Frontline Tri-Act^®^/Frontect^®^ kills fleas before they lay eggs, thus preventing environmental contamination by immature flea stages.

## Conflict of interest

This clinical study was funded by Merial S.A.S., 29 Avenue Tony Garnier, 69007 Lyon, France of which Frédéric Beugnet, Lénaïg Halos and Wilfried Lebon are employees.

ClinVet, of which the other co-authors are employees, is an independent South African Contract Research Organisation contracted to conduct the study.

All authors voluntarily publish this article and have no personal interest in these studies other than publishing the scientific findings that they have been involved in via planning, initiating, monitoring and conducting the investigations and analysing the results.
